# Current Topics of Progressive Cardiac Conduction Disease

**DOI:** 10.1002/joa3.70383

**Published:** 2026-06-25

**Authors:** Naokata Sumitomo, Hitoshi Mori, Takashi Kumamoto, Taisuke Ishikawa, Akiko Seki, Naomasa Makita, Jean‐Jacques Schott, Arthur A. M. Wilde

**Affiliations:** ^1^ Department of Pediatric Cardiology Saitama Medical University International Medical Center Saitama Japan; ^2^ Department of Cardiology Saitama Medical University International Medical Center Saitama Japan; ^3^ Department of Pediatrics Saga University Saga Japan; ^4^ Veterinary Teaching Hospital, Joint Faculty of Veterinary Medicine Kagoshima University Kagoshima Japan; ^5^ Department of Cardiology Tokyo Women's Medical University Tokyo Japan; ^6^ Department of Preventive Medicine Tokyo Women's Medical University Tokyo Japan; ^7^ Department of Cardiology Sapporo Teishinkai Hospital Sapporo Japan; ^8^ Nantes Université CNRS, INSERM, L'institut du Thorax Nantes France; ^9^ ERN GUARD‐Heart Amsterdam the Netherlands; ^10^ Department of Cardiology Amsterdam University Medical Centre Amsterdam the Netherlands

**Keywords:** implantable cardioverter defibrillator, inherited arrhythmia, pacemaker implantation, progressive cardiac conduction disease, sudden cardiac death

## Abstract

Progressive cardiac conduction disease (PCCD) is a rare inheritable cardiac conduction system disease that may lead to complete heart block, syncope, or sudden cardiac death. The estimated incidence of PCCD is 0.0005%/year. Recent advances in genetics have revealed a wide variety of diseases associated with the development of PCCD. Ion channel genes (*SCN5A, KCNK17, TRPM4, HCN4*), gap junction genes (*GJA5*, *GJC1*), genes associated with myopathy (*LMNA*, *EMD*, *DES*, *DMD*, *TNNI3K*, *DMPK, ZNF9*), transcription factor genes (*NKX2‐5*, *TBX5*), genes associated with metabolic disease (*PRKAG2*, *LAMP2*, *GLA*), and mitochondrial gene variants have all been associated with the occurrence of PCCD. Pacemaker implantation is the first‐line therapy for PCCD, but implantable cardioverter defibrillator implantation should be considered in some cases. This review aims to provide a clinically relevant overview of these different forms, with emphasis on their genetic basis and phenotypic expression. We propose a practical classification distinguishing isolated and associated forms of PCCD and discuss the implications for diagnosis, risk stratification, and patient management.

## Graphical abstract

Many genes and the protein cause PCCD. Mutation of Na_V_1.5 or CX40 cause isolated PCCD, but mutation of lamin A/C, emerin, or desmin lead to cardiomyopathy, and PCCD. Mutation of transcription factor NCX2‐5, and Tbx5 associated with atrial septal defect and abnormal development of conduction system. Abnormal substance deposition inside the cell may leads to myocardial disease and PCCD. Mitochondria disease cause decrease of cellular energy (ATP) production, cellular damage and fibrosis.

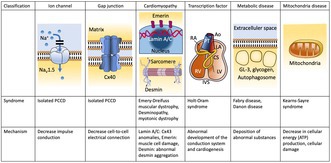

## 1 Introduction

Progressive cardiac conduction disease (PCCD), previously synonymous with Lenègre [1] or Lev [2] disease, is a progressive cardiac conduction system disturbance in the absence of any structural heart disease that may lead to complete heart block, syncope, or sudden cardiac death. The work of Lenègre [1] and Lev [2] was based on the pathological finding that the destruction of the His bundle and the bundle branches is the main finding in atrioventricular block. Familial inheritance of cardiac conduction disease [3] has already been reported, and the progress of the destruction of the cardiac conduction system [4, 5] has been reported thereafter, and PCCD is recognized as one of the primary electrical heart diseases such as long QT syndrome (LQTS) and Brugada syndrome (BrS).

PCCD was first reported to affect patients without any structural or congenital heart disease that could cause complete heart block, including ischemic heart disease, cardiac tumors, myocarditis, or cardiac surgery, but with the recent progress in genetic analyses, those with familial PCCD are usually associated with genetic variants of cardiac ion channels or gap junctions, whereas patients with cardiomyopathy, congenital heart disease, metabolic disease, or syndrome with systemic disease may have some extent of a progressive conduction disturbance. Those patients are usually associated with variants of genes encoding transcription factors, enzymes, or cardiac structural proteins.

The current definition of PCCD may now be insufficient. It is traditionally defined as a degenerative disorder of the His–Purkinje system leading to conduction block in the absence of structural heart disease. In this review, we defined PCCD as a degenerative disorder or progressive malfunction of the AV node, His‐Purkinje system, and familial sick sinus, or familial atrial fibrillation were excluded. This review will use a genotype‐based subdivision of PCCD according to recent topics and advancements in the study of PCCD.

## 2 Incidence

**FIGURE 1:**
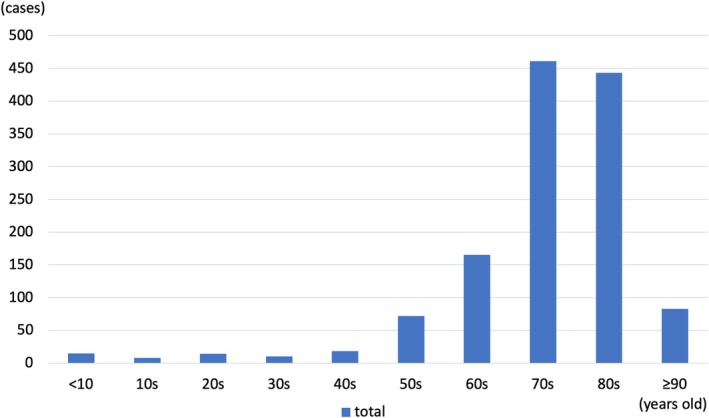
Age‐related differences in the number of pacemaker implantations for AV block. The number of PMIs for cAVB, paroxysmal AVB, Mobitz type II block, and tri‐fascicular block at Saitama Medical University International Medical Center are indicated in this figure. PMIs for AV block increased after 50 years old.

The precise incidence of PCCD is unknown. However, the incidence of pacemaker implantation without obvious heart disease increases with age, therefore, PCCD has been considered part of the aging process. The incidence of pacemaker implantation for complete atrio‐ventricular (AV) block, paroxysmal AV block, Mobitz type II AV block, and tri‐fascicular block in our hospital is shown in Figure 1. Apparently, pacemaker implantations (PMIs) for AV block increase with age, and rarely occur before age 50 (65 of 1289 PMIs, 5%) and become common after age 50 (95%). It has been reported that 0.21%–2.28% of patients undergoing PMI are suspected to have PCCD [6]. According to the report of the Japan Arrhythmia Industry Association (https://www.jadia.or.jp/index.html), the total number of new single and dual chamber PMIs in Japan during 2008 was 32 985 cases. On the assumption that the incidence of PCCD is 2% of PMIs in Japan, the estimated number of PMIs for PCCD in Japan would be 660 cases/year (0.0005%/year).

The incidence of PCCD in children is also unknown. From heart screening data in Tokyo (Tokyo Health Service Association, 2008–2023, https://www.yobouigaku‐tokyo.or.jp/nenpo/), complete heart block was detected in 5/754141 (0.001%), 5/606862 (0.001%), and 6/72824 (0.008%) of 1st‐, 7th‐, and 10th‐grade students, respectively, indicating that conduction disturbances may also have increased in children. However, not all complete heart block children suffered from PCCD. If the proportion of PMIs was the same as in our hospital and the incidence of PCCD was the same in children, the estimated PMIs for PCCD would be 7.7 (0.00007%/year) in those aged < 10 years and 4 (0.000034%/year) in those aged 10–19 years.

## 3 Epidemiology

The true incidence of PCCD remains unknown. Current estimates are largely derived from pacemaker implantation data, which introduces bias.

It may be worth emphasizing that PCCD is likely:

- underdiagnosed in younger populations
- frequently misclassified as a degenerative disease
- strongly age‐dependent

Therefore, epidemiological data should be interpreted as reflecting an approximate burden rather than true prevalence.

## 4 Inheritance

Most cases of congenital and inherited PCCD follow an autosomal dominant inheritance pattern, although some cases are autosomal recessive or sporadic [7, 8, 9]. An X‐linked recessive or X‐linked dominant form has also been reported in PCCD associated with myopathy (see *DES*, *DMD*, and *LAMP2*). The inheritance patterns of mitochondrial diseases are known to be maternal mitochondrial DNA inheritance, nuclear DNA autosomal dominant or recessive inheritance, or sporadic.

## 5 Electrocardiographic Features

The typical electrocardiographic features of PCCD include a combination of PR interval prolongation and QRS prolongation over several years, and QRS axis deviation due to delayed conduction and/or block within or between the AV node, His–Purkinje system, or ventricles. These findings are not present at an early stage, but develop progressively with age [10]. Usually, the AV block pattern is type II (not Wenckebach type), paroxysmal, or complete heart block, and the HV interval is prolonged. In cases associated with an *SCN5A* variant, a BrS type ST‐T change may be present [11].

## 6 Clinical Features

Typical clinical features of PCCD are recurrent syncope or sudden cardiac death due to cardiac arrest or bradycardia, and sometimes due to ventricular fibrillation [12]. If patients develop PCCD in association with cardiomyopathy, congenital heart disease, metabolic disease, or a systemic disease syndrome, the clinical manifestations of the underlying disease are present. Ventricular arrhythmias may occur independently of bradycardia, which has important clinical implications.

## 7 Genetic Classification of PCCD

The 2022 genetic testing document states that, as a Class I indication, targeted genetic testing is recommended as part of the diagnostic evaluation for index patients with isolated cardiac conduction disease (CCD/PCCD) or with concomitant structural heart or extracardiac disease, particularly in those with a diagnosis of early‐onset disease or suspected laminopathy, especially when there is documentation of a positive family history of CCD/PCCD [13]. Four genes (*SCN5A*, *LMNA*, *GLA*, and *PRKAG2*) are recommended to be investigated [13]. Based on recent advancements in genetic testing, we propose several disease groups that cause PCCD [14, 15].

### 7.1 Isolated PCCD (Primary Electrical Disease)

#### 7.1.1 Ion Channel Disease

##### 7.1.1.1 SCN5A

**FIGURE 2:**
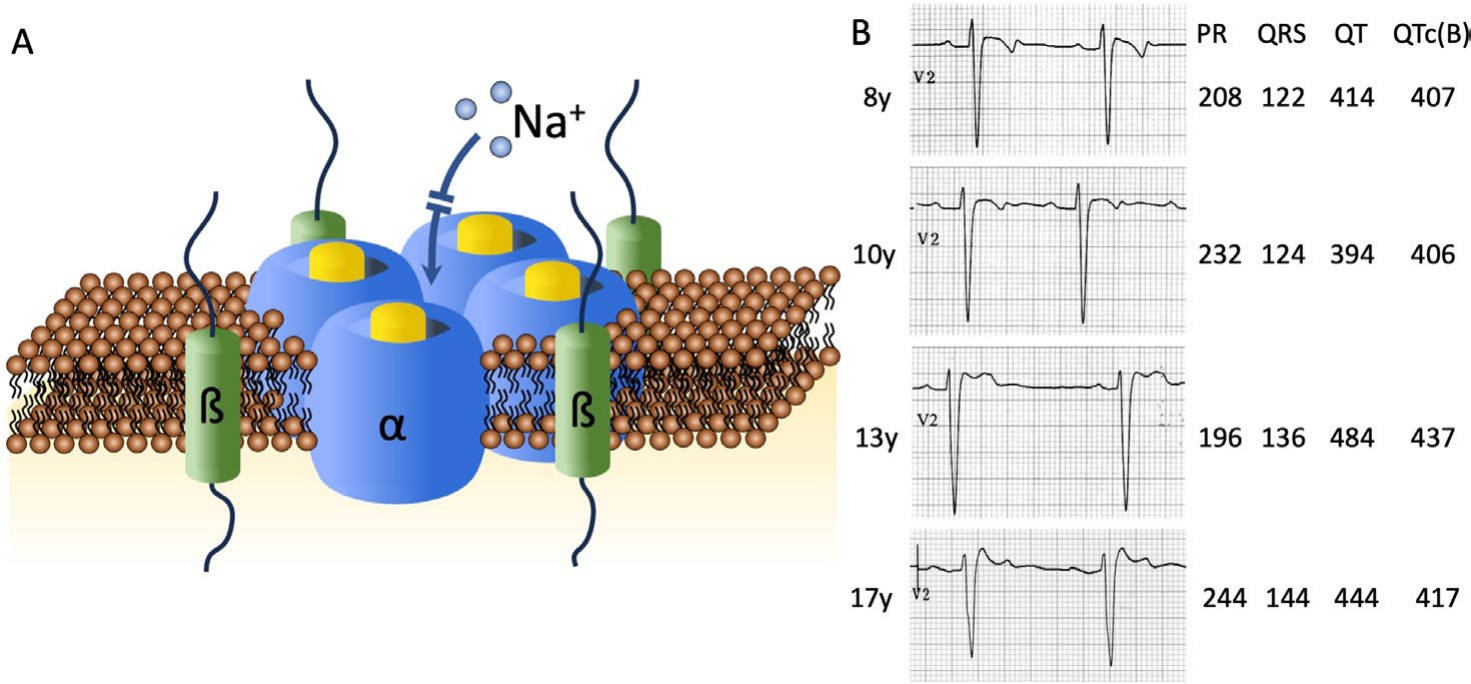
Structure of voltage‐gated cardiac sodium channels and the time course of the electrocardiogram in a patient with Brugada syndrome. (A) Structure of the voltage‐gated cardiac sodium channel (NaV1.5): The α subunit was encoded by the *SCN5A* gene and the ß‐subunit was encoded from the *SCN1B* gene. (B) Serial change in the electrocardiogram. The time course of the ECG changes in a boy with Brugada syndrome (BrS) revealed a gradual prolongation of the QRS duration and QT interval. Genetic analysis revealed an *SCN5A* gene variant (Q55X). This case was not defined as a classical form of PCCD, but the development of a conduction disturbance may complicate some patients with BrS.

The *SCN5A* gene encodes the α subunit of voltage‐gated cardiac sodium channels (Na_V_1.5) (Figure 2A), which mediates the inward sodium current (I_Na_) responsible for impulse conduction inside and between contractile myocytes and the specialized conduction system. Gain of function variants in *SCN5A* result in an increase in the late sodium inward current, I_Na late_, which is well known to cause LQTS type 3 [16]. In contrast, loss‐of‐function *SCN5A* variants decrease the peak I_Na_ and cause BrS [17], idiopathic ventricular fibrillation [18], sick sinus syndrome [7], and/or PCCD [19, 20]. Overlap syndromes with sick sinus syndrome, BrS, and ventricular tachycardia that are mostly due to loss‐of‐function variants of the *SCN5A* gene have also been reported [21]. The PR interval and QRS duration are longer in children [22] and adults [23] with BrS and pathogenic or likely pathogenic *SCN5A* gene variants than in those without *SCN5A* gene variants (Figure 2B). It has also been reported that after puberty in girls with BrS, the Brugada ECG pattern reappears, whereas the bradycardic aspect of the disease persists or even worsens [22]. In addition, female patients with familial BrS further may develop sick sinus syndrome, which has not been observed in male patients [24].

##### 7.1.1.2 KCNK17

A variant in the potassium two pore domain channel subfamily K member 17 (*KCNK17*) gene encoding the pH‐sensitive cardiac two‐pore domain potassium channel (K2P) TASK‐4 has been identified as a contributor to a progressive and severe cardiac conduction disorder combined with idiopathic ventricular fibrillation. Gain‐of‐function of TASK‐4 in the conduction system leads to hyperpolarization and marked slowing of the upstroke velocity in spontaneously beating cells, which may further aggravate conduction slowing associated with loss of sodium channel function [25].

##### 7.1.1.3 TRPM4

Transient receptor potential cation channel subfamily M member 4 (*TRPM4*) is a Ca^2+^‐activated non‐specific channel that mediates transport of monovalent cations across membranes, thereby depolarizing the membrane. A gain of function variant of *TRPM4* has been identified in PCCD families [26]. A loss‐of‐function *TRPM4* variant downregulates the mRNA levels of *HEY2*, *TBX5*, and *NKX2.5* transcription factors associated with the inhibition of conduction system development and the ventricles in human induced pluripotent stem cell‐derived cardiomyocytes [27]. The precise mechanism of the occurrence of the *TRPM4* variant has not been reported, but gain of function variants may depolarize conduction system cells, reduce the availability of cardiac sodium channels and currents, and thereby alter the normal impulse propagation in Purkinje fibers. Conversely, loss‐of‐function variants of *TRPM4* may lead to hyperpolarization of the membrane potential and therefore reduce the cellular excitability and conduction [28].

##### 7.1.1.4 HCN4

The hyperpolarization‐activated cyclic nucleotide‐gated (HCN) cation (Na^+^/K^+^) current member 4 (HCN4) is known to play a major role in the automaticity current of the sinus node through slow phase 4 diastolic depolarization. The *HCN4* gene is expressed throughout cardiac tissue, and *HCN4* variants have been reported with a broad spectrum of phenotypes including sick sinus syndrome [29], inappropriate sinus tachycardia, early‐onset AF, AV block [30], idiopathic ventricular tachycardia (VT), and non‐compaction of the left ventricle (LVNC) [31].

#### 7.1.2 Gap Junction

##### 7.1.2.1 GJA5

**FIGURE 3:**
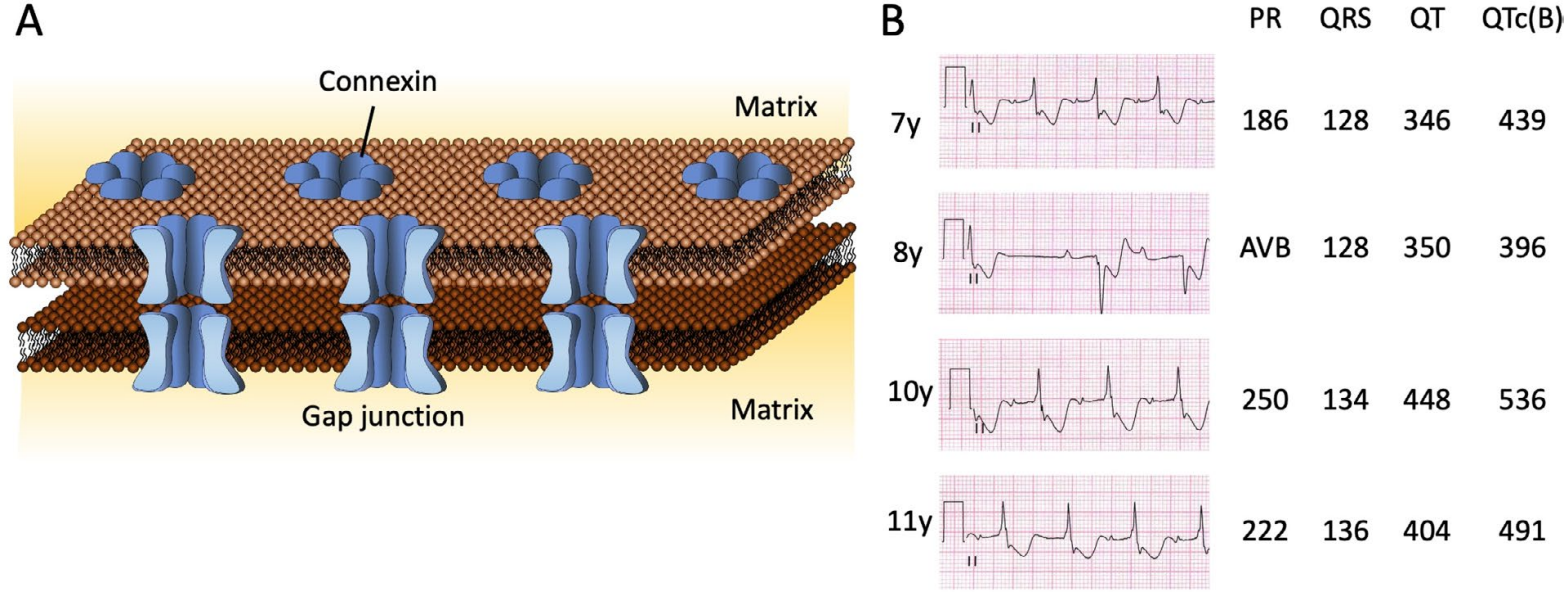
Structure of Gap junctions and serial electrocardiograms in a PCCD patient with a *GJA5* gene variant (Q58L). (A) Structure of Gap junctions between adjacent cells. (B) A 6‐year‐old boy was referred to our clinic for left axis deviation and left bundle branch block detected during heart screening, and was followed up for mild cardiomegaly (CTR 0.5). He suddenly died at 11 years‐old, and his mother died suddenly after delivery of his sister when she was 30 years old. Serial ECGs showed progressive prolongation of the PR interval, QRS duration, and QT interval. Genetic testing of the proband and his sister revealed a *GJA5* gene variant (Q58L), which causes a connexin 40 variant in the gap junctions of the myocardium [32].

Gap junctions consist of a unit, composed of 6 of the same membrane protein (Connexin) (Figure 3A), between cells, and function as cell‐to‐cell ion channels and to exchange small molecules such as cyclic adenosine monophosphate (cAMP), adenosine triphosphate (ATP), and inositol triphosphate (IP3). They function as electrical connections between cardiac myocytes and specialized conduction myocytes to co‐ordinate the conduction (and contraction) of the heart and are involved in excitation‐contraction coupling. *GJA5* encodes connexin 40 (Cx40), which is expressed mainly in the atrium and His–Purkinje system and in the ventricle in the early development of the heart. *GJA5* variant causes an early onset of PCCD, bundle branch block, and sudden cardiac death [32] (Figure 3B).

##### 7.1.2.2 GJC1

*GJC1* encodes the Cx45, that forms voltage‐sensitive channels with very low conductance and is mainly found in the AV node and adjoining His bundles. Variants of *GJC1* are associated with progressive atrial conduction defects with craniofacial and dentodigital malformation [33].

### 7.2 Syndromic/Secondary PCCD

#### 7.2.1 Cardiomyopathies

##### 7.2.1.1 LMNA

**FIGURE 4:**
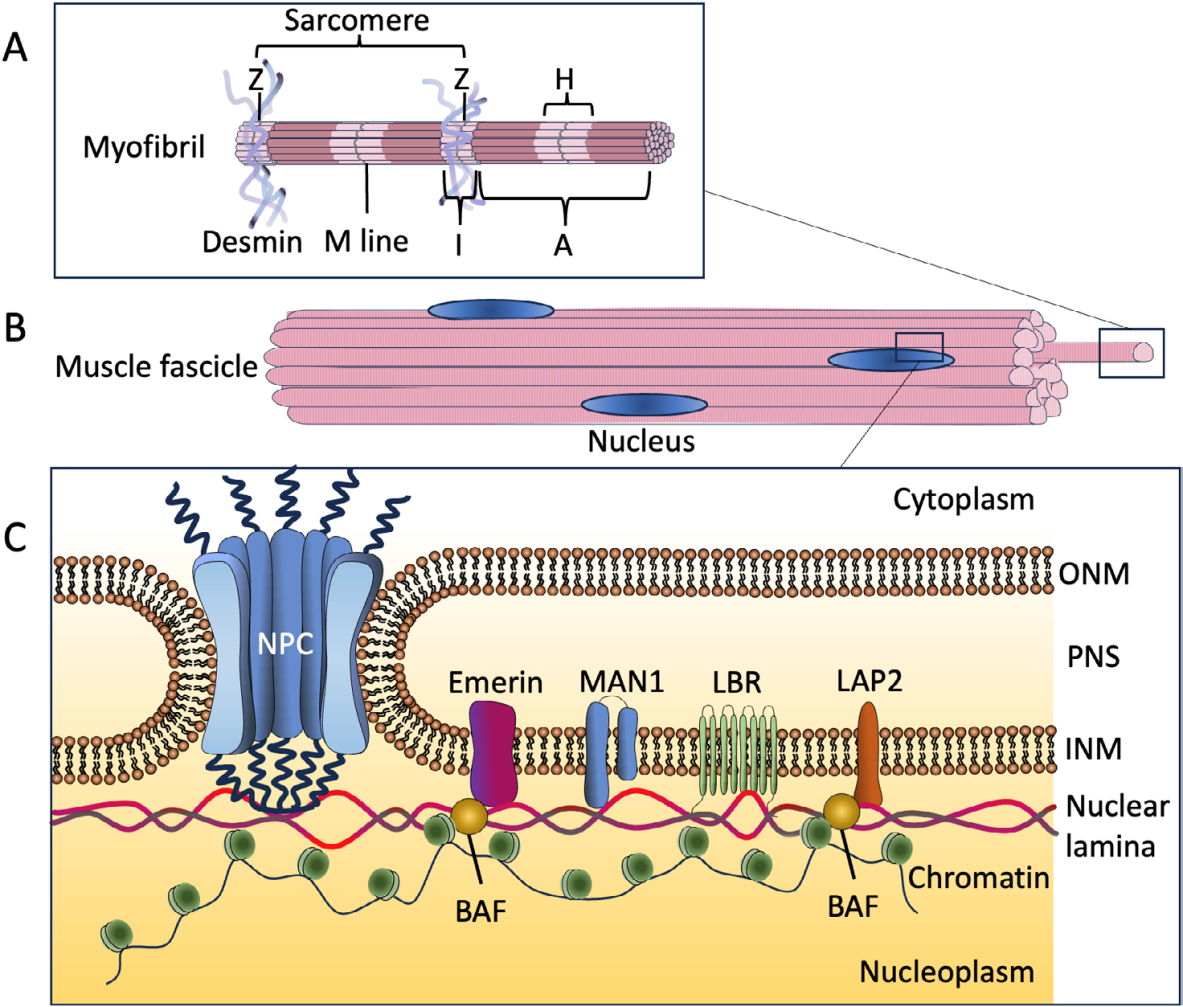
Structure of myocardium. (A) Structure of the myofibrils. (B) Structure of myocardial fascicles. (C) Interactions among nuclear envelope proteins, the nuclear lamina, and chromatin. Z = Z‐band; H = H‐band; I = I‐band; A = A‐band; NPC = nuclear pore complex; INM = inner nuclear membrane; ONM = outer nuclear membrane; PNS = perinuclear space; MAN1 = mannosyl‐oligosaccharide 1; LAP2 = lamina‐associated polypeptide 2; SUN1/2 = Sad1p, UNC‐84 domain protein 1 and 2; LBR = Lamin B Receptor.

**FIGURE 5:**
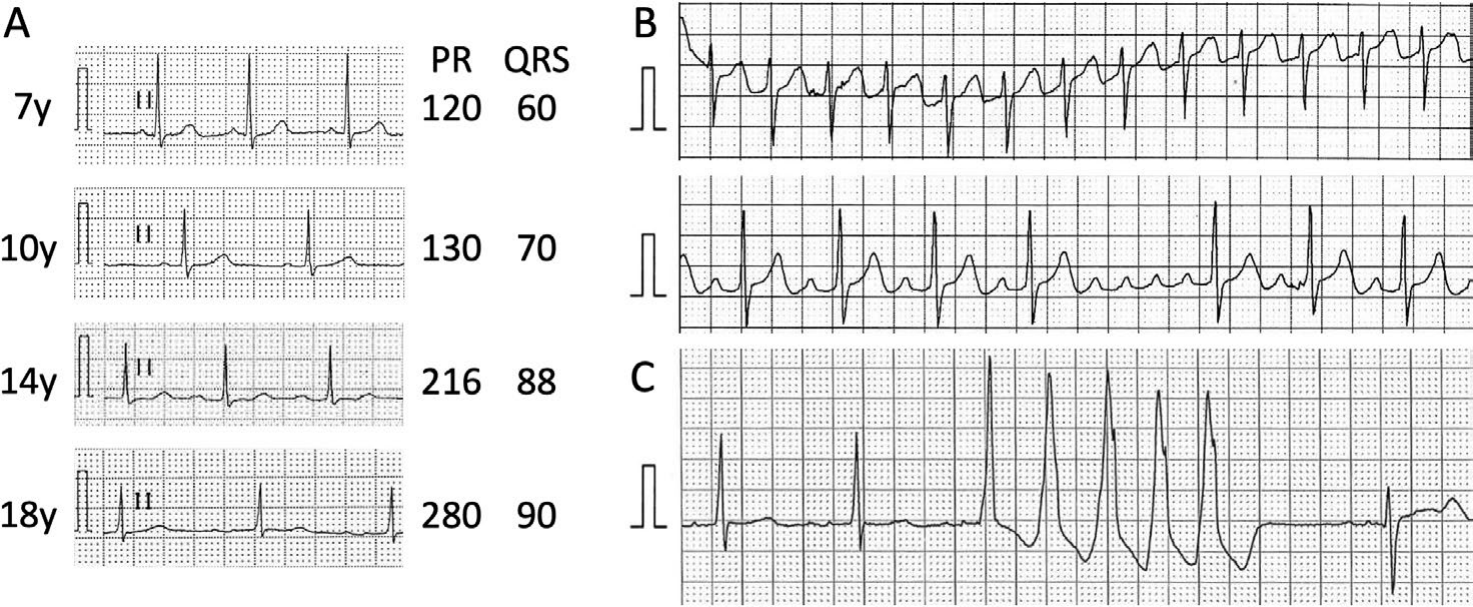
ECG of a patient with Emery‐Dreifuss muscular dystrophy. When she was 3‐years‐old, it was noticed she became slower when running and started to climb stairs one step at a time while holding her knee. Grower's sign was positive from 5‐years‐old and genetic testing revealed an de novo heterozygous *LMNA* mutation (c.810+1G〉T). She was diagnosed with Emery‐Dreifuss muscular dystrophy when she was 9‐years‐old. She underwent catheter ablation of atrial tachycardia and atrial flutter when she was 12‐years‐old. During the electrophysiological study, her AH interval was 154 ms and HV 56 ms. (A) Serial ECG change: Serial electrogram showing progressive prolongation of the PR interval and a decrease in the P wave and R wave amplitudes. (B) Atrial flutter: Frequent episodes of atrial flutter were documented by Holter electrocardiogram when she was 12‐years‐old. (C) Nonsustained ventricular tachycardia: Ventricular arrhythmias were recorded from 17‐years‐old.

Lamins are intermediate filament proteins that make up the structural element of nuclear lamina, and they not only support the structure of the nucleus, but also influence the regulation of gene expression through an interaction with transcription factors, DNA, and chromatin [34] (Figure 4C). Pathogenic variants of the lamin A/C gene (*LMNA*) are associated with multisystem disease or laminopathy, including Emery‐Dreifuss muscular dystrophy Type 2 (Figure 5) and 3, limb‐girdle muscular dystrophy, Hutchinson‐Gilford progeria syndrome, Werner's syndrome, and Charcot–Marie–Tooth disease Type 2B1. Cardiac involvement associated with *LMNA* variants, including dilated cardiomyopathy and PCCD, has been shown to be due to gap junction anomalies of Cx43, which has been shown to connect the working myocardium in mouse models [35].

##### 7.2.1.2 EMD

The EMD (emerin) gene encodes the nuclear membrane protein emerin (Figure 4C), and mutations in EMD cause Emery‐Dreifuss muscular dystrophy type 1. This disease is inherited in an X‐linked recessive pattern. Emerin functions in nuclear‐cytoskeletal linkage, chromatin remodeling, transcription regulation, and signal transduction. Mutations of emerin in the specialized conduction system cause loss of nuclear envelope stability and rupture, which may lead to muscle cell damage and fibrosis. Both Emery‐Dreifuss muscular dystrophy type 1 and type 2 are associated with a high risk of progressive conduction system disease, sudden cardiac death due to ventricular arrhythmias, and dilated cardiomyopathy [36].

##### 7.2.1.3 DES

**FIGURE 6:**
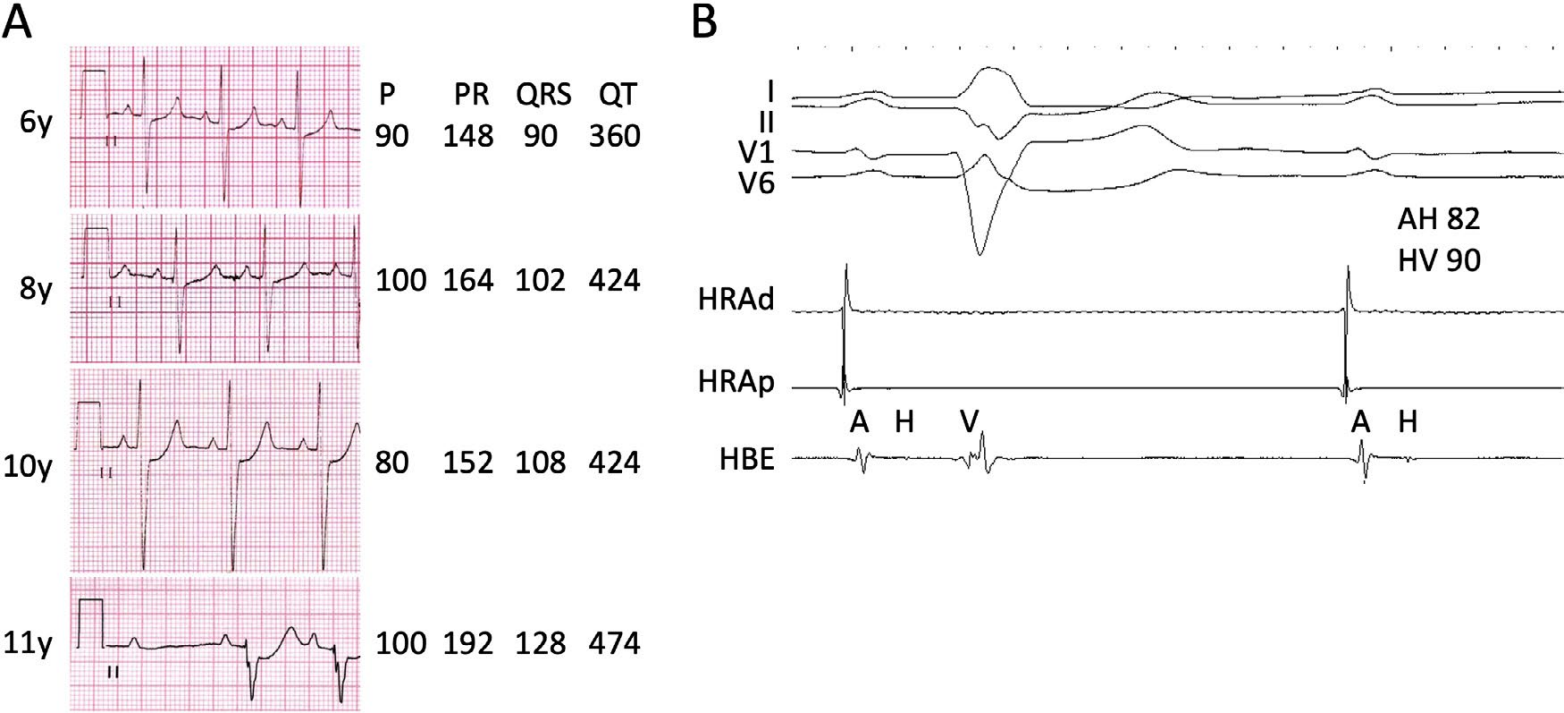
Electrocardiogram in a patient with desminopathy [35]. She was followed up for left axis deviation and an interventricular conduction disturbance from 6‐years‐old. She complained of a headache and dizziness due to paroxysmal heart block, and a pacemaker was implanted. Genetic testing revealed a variant of the *DES* gene (R454W), suggesting desminopathy as the cause of her disease [37]. (A) Serial electrocardiograms before pacemaker implantation Serial ECGs showed progressive prolongation of the PR interval and QRS duration. (B) Intracardiac electrocardiogram Electrophysiological study showed HV prolongation (AH 82 ms, HV 90 ms) and HV block.

Desmin is a muscle‐specific intermediate filament protein that connects desmosomes, mitochondria, and Z‐bands to the cytoskeleton (Figure 4A). Variant in the *DES* gene that disrupt the cytoplasmic desmin network, cause an accumulation of abnormal desmin aggregation, thus causing skeletal myopathies and a variety of cardiomyopathies (desminopathies) (Figure 6), including restrictive cardiomyopathy, dilated cardiomyopathy (DCM), LVNC, conduction disturbances [37], and ventricular arrhythmias.

##### 7.2.1.4 DMD

Mutations in the dystrophin gene, which encodes a structural cytoskeletal protein, cause Duchenne muscular dystrophy (DMD), Becker muscular dystrophy (BMD), X‐linked dilated cardiomyopathy, and cardiomyopathy in female carriers of DMD and BMD [38]. The classical ECG pattern findings include tall R waves and an increased R/S ratio in lead V1, Q waves in the left precordial leads, right axis deviation, and complete right bundle branch block due to fibrosis in the basal posterior wall of the heart and may reflect reduced electrical activity in the inferobasal ventricular wall. Pathological examination of the hearts of dystrophic patients has demonstrated fibrosis involving the conduction system in addition to the myocardium.

##### 7.2.1.5 TNNI3K

Cardiac troponin‐I interacting MAP kinase (TNNI3K) is an important kinase in cardiac morphogenesis and sarcomere organization. A rare genetic variant of the *TNNI3K* gene has been linked to supraventricular tachycardia, conduction disturbances, and DCM [39]. Although the precise mechanism is unknown, there has been a positive correlation between the *TNNI3K* mRNA level and PR interval as demonstrated in mice models [40].

##### 7.2.1.6 DMPK

Variant of the myotonic dystrophy protein kinase gene (*DMPK*) causes a common form of myotonic dystrophy (DM1) by an expansion of cathepsin G (CTG) trinucleotide repeating during cell division [41]. DM1 is a multisystemic disease that includes myotonia, progressive muscle weakness, cataracts, diabetes mellitus, mental retardation, and hypogammaglobulinaemia [41]. Cardiac involvement of DM1 results in LV hypertrophy and dilatation, systolic dysfunction, mitral valve prolapse, and left atrial dilatation. Slowly progressive conduction disturbances are common in DM1 patients, resulting from RNA toxicity‐induced overexpression of NKX2‐5 and subsequent down‐regulation of Cx40 and Cx43 as demonstrated in transgenic mouse models [42]. It is also reported that expanding CUG repeats by mutant RNAs leads to muscleblind‐like1 (MBNL1) proteins that reproduce splicing alteration of *SCN5A* mRNA, reduced excitability, and may produce cardiac‐conduction disturbance [43]. SCD developed in 18 of 506 (3.6%) Japanese patients with DM1. The incidence of VT was 0.2%, whereas increased QRS duration (QRS ≥ 120 m) and prolonged PQ interval (PQ ≥ 120 ms) were observed in 20.3% and 11.8% of patients, respectively; however, these parameters were not significantly associated with the occurrence of SCD. PMI was associated with SCD, with a hazard ratio of 4.35 (95% CI, 1.22–15.50) on univariate analysis. These findings suggest that ICD implantation or cardiac resynchronization therapy may help prevent SCD in selected patients with DM1 [44].

##### 7.2.1.7 ZNF9

In DM2, a CCTG repeat expansion occurs within intron 1 of the zinc finger protein 9 (*ZNF9*) gene [14]. Although DM2 has been less extensively investigated, patients with DM2 also exhibit an increased incidence of PR interval prolongation and evidence of heart block [45].

#### 7.2.2 Transcription Factors

##### 7.2.2.1 NKX2‐5

**FIGURE 7:**
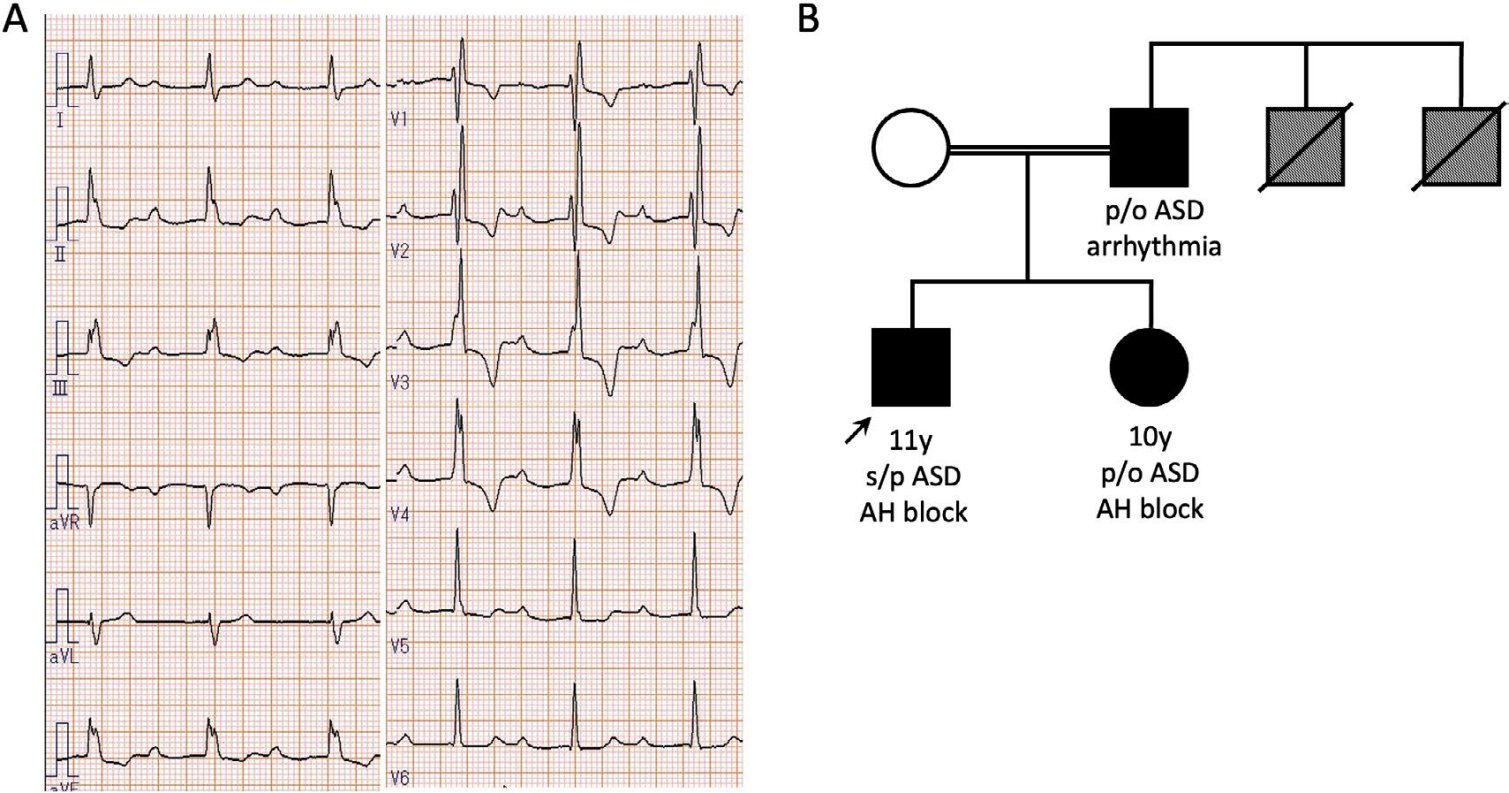
Congenital heart disease and conduction disturbances. (A) Electrocardiogram of the proband. A 6‐year‐old boy was referred to outpatient clinic for 1st degree AV block and incomplete right bundle branch block detected during heart screening. He was previously diagnosed with a secundum atrial septal defect (ASD) that naturally closed during follow up. His AH interval was 332 ms and HV 54 ms. (B) Family tree of the index patient. His family history was remarkable in that his sister had a repaired ASD and AV block, his father a repaired ASD when he was 11 years old, and the brothers of his father died during infancy with unknown origin heart disease.

NK‐2 transcription factor related, locus 5 (*NKX2‐5*) is a homeobox transcription factor, and the *NKX2‐5* variant causes secundum atrial septal defects and AV block after birth [46]. *NKX2‐5* ^+/−^ mice are reported to express only the Cx40^+^/Cx45^+^ compartment in the AV nodal region, which is important for connection to the His bundle, but lack the Cx40^−^/Cx45^+^ compartment that is essential for AV node development [46]. Consequently, *NKX2‐5* ^+/−^ mice develop small AV nodes, but have normal development below the His bundle, and the HV interval stays in the normal range, but the AH interval prolongs as they grow up [47]. A familial atrial septal defect (ASD) with prolongation of the PR interval, strongly suggestive of an *NKX2‐5* variant, is shown in Figure 7.

##### 7.2.2.2 TBX5

Variant of the T‐box transcription factor gene *TBX5* causes Holt‐Oram syndrome, and patients with this nonsense or splicing variant exhibit secundum atrial septal defects, progressive AV block, and radial deformities of the upper limb [48]. It has been reported that heterozygous *TBX5* ^*−/+*^ mice exhibit a reduced expression of CX40 and Na_V_1.5, resulting in a loss of fast conduction of the AV bundle and bundle branches, and a prolonged PR interval, His duration, HV interval, and QRS duration [49].

#### 7.2.3 Metabolic Diseases

##### 7.2.3.1 PRKAG2

Variants in the gene encoding the gamma2 subunit of the adenosine monophosphate (AMP)‐activated protein kinase gene (*PRKAG2*) may cause left ventricular hypertrophy resembling hypertrophic cardiomyopathy (HCM), Wolff–Parkinson‐White syndrome, and AV block. Mutant PRKAG2 promotes deposition of a glycogen‐like substance, amylopectin, in the heart that leads to the development of HCM, accessory pathways, and PCCD [50].

##### 7.2.3.2 LAMP2

Variant of lysosomal‐associated membrane protein 2 (*LAMP2*) produces autophagic vacuolar myopathies (Danon disease), a rare X‐linked dominant disorder. Clinical manifestations of Danon disease are progressive muscular dystrophy, mental retardation, pigmentary retinopathy, LV hypertrophy, accessory pathways, and conduction disturbances. Conduction disturbances arise from infiltration and pathological alterations in the conduction system in a mice model [51].

##### 7.2.3.3 GLA

Fabry disease is an X‐linked lysosomal storage disease caused by deficiency of the enzyme α‐galactosidase A due to variants in the galactosidase alpha gene (*GLA*) [52], affecting multiple organ systems including the heart, nervous system, gastrointestinal system, and kidneys. Clinical cardiac manifestations include myocardial hypertrophy, restrictive cardiomyopathy, ST depression, T‐wave elevation in the left precordial leads, and a prolonged PR interval and QRS duration [53].

#### 7.2.4 Mitochondria Disease

**FIGURE 8:**
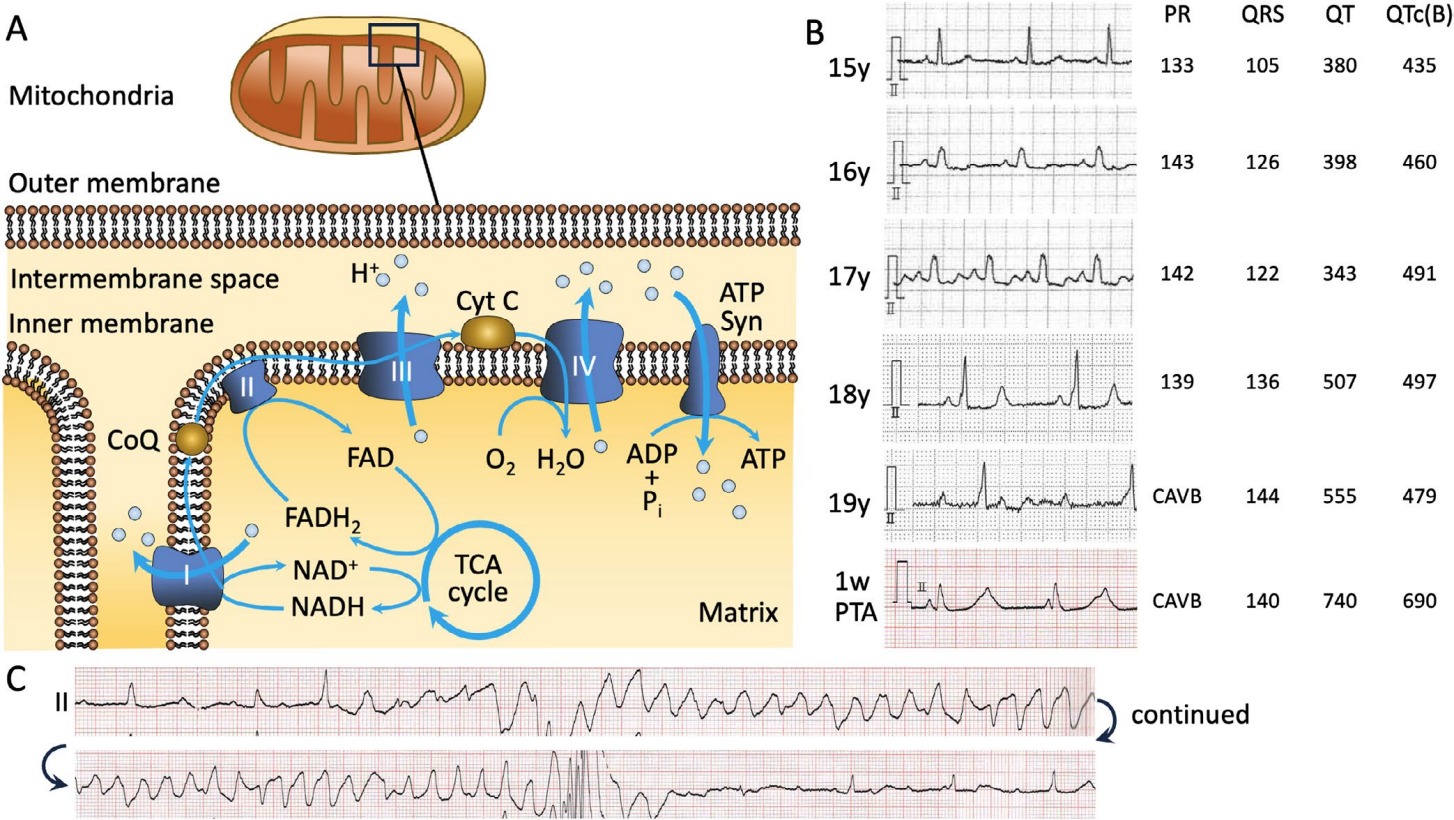
Mitochondria disease. (A) Electron transport chain in mitochondria. I = complex I (NADH–ubiquinone oxidoreductase); II = complex II (succinate–ubiquinone reductase); III = complex III (coenzyme Q‐cytochrome c oxidoreductase); IV = complex IV (cytochrome C oxidase); ADP, adenosine diphosphate; ATP Syn, ATP synthase (complex V); ATP, adenosine triphosphate; CoQ, coenzyme Q (ubiquinone); Cyt C, cytochrome C; FAD, flavin adenine dinucleotide; FADH_2_: Reduced form of flavin adenine dinucleotide; NAD^+^, nicotinamide adenine dinucleotide (oxidized form); NADH, reduced form of nicotinamide adenine dinucleotide; Pi, inorganic phosphate; TCA cycle, tricarboxylic acid cycle. (B) Serial ECG changes in Kearns–Sayre syndrome [12]. Serial ECGs show a gradual increase in the PR interval and QRS duration, and a markedly prolonged QT interval. CAVB, complete AV block; PTA, prior to admission. (C) Development of torsade de pointes.

Mitochondria provide the energy to organs throughout the body through ATP production (Figure 8A), therefore, mitochondrial dysfunction results in diseases affecting multiple organs including the brain, nerves, skeletal and cardiac muscle, and the endocrine system disease. Partial or multiple deletion of mitochondria DNA (mtDNA) causes Kearns–Sayre syndrome. Conduction disease is most commonly observed in Kearns‐Sayre syndrome [12], including complete AV block, chronic progressive external ophthalmoplegia, ataxia, and pigmentary retinal degeneration. Serial ECGs indicate a progressive PR interval, QRS duration, and QT interval (Figure 8B), and then the development of torsade de pointes in a case of Kearns‐Sayre syndrome [12] (Figure 8C).

**FIGURE 9:**
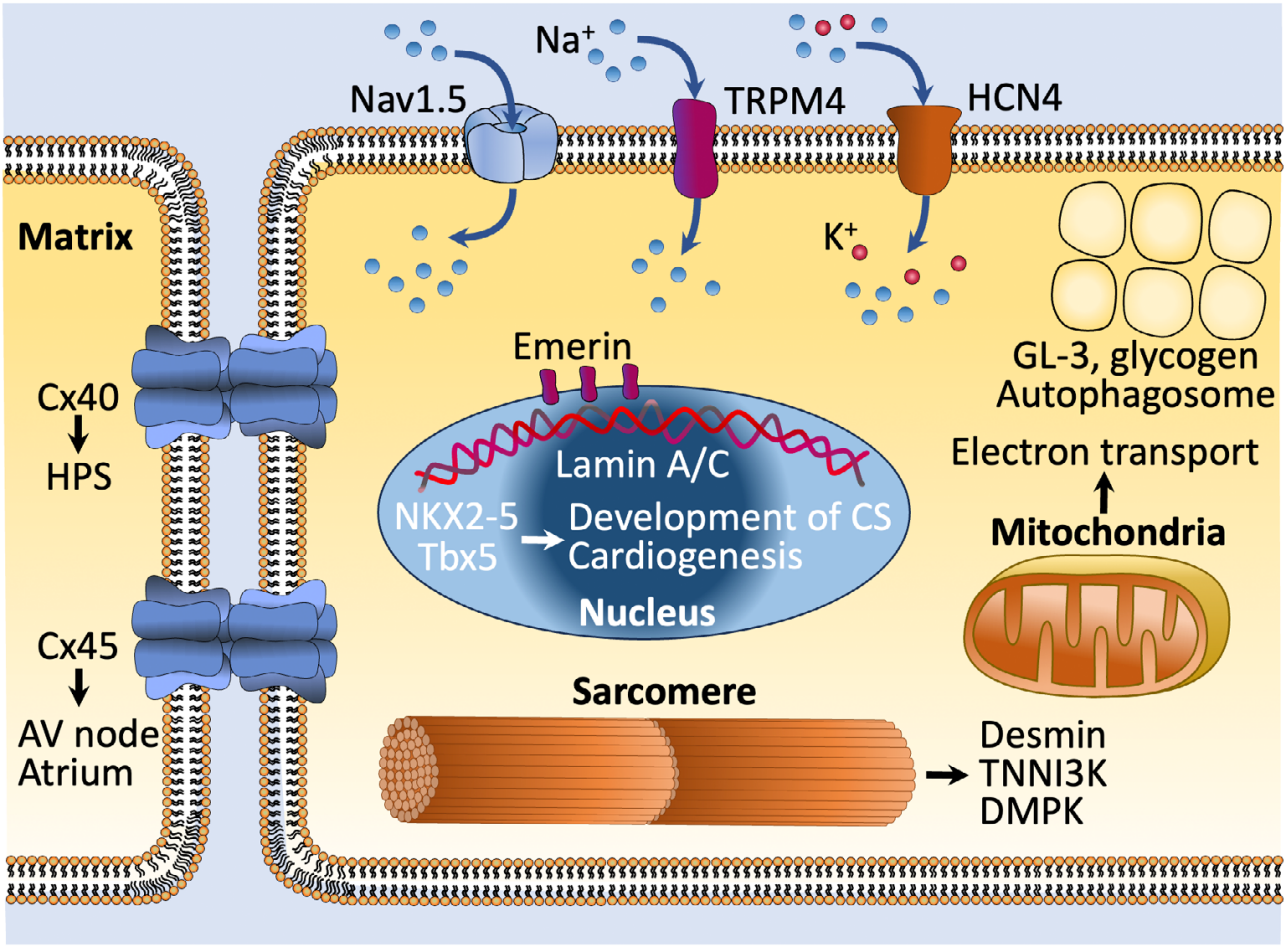
Possible mechanisms to cause PCCD. The main mechanisms to cause PCCD were shown in this figure. Variants in the ion channels (Na_V_1.5, TRPM4, HCN4), gap junctions (Cx40 and Cx45), sarcomeres (Desmin, TNNI3K, and DMPK), nuclear lamina (lamin A/C), nuclear membrane protein (Emerin), transcription factors (NKX2‐5 and Tbx5), mitochondria electron transport, and cell storage disease (GL‐3 and glycogen) and autophagic vacuolar myopathies were reported as causes of PCCD. DMPK, dystrophia myotonica‐protein kinase; CS, conduction system; GL‐3, globotriaosylceramide; HPS, His Purkinje system.

The possible mechanisms that may lead to PCCD are shown in Figure 9.

## 8 Genetic Architecture of PCCD

According to the mechanism, clinical features, and associated risk, we propose these genes be classified into 3 grades (Table 1).

**TABLE 1:** Genes associated with PCCD.

Gene	Protein	Inheritance	Other syndromes or diseases	Mechanism	Risk	ClinGen classification
1. Ion channel						
*SCN5A*	Na_V_1.5	AD, AR	BrS, LQT3, DCM, overlap syndrome	Na channel LOF	High	NA/major gene for SSS; definite for LQTS, BrS
*KCNK17*	TASK‐4	AD	LQT2, Ischemic stroke, cerebral hemorrhage, Ischemic and nonischemic HF, AF, VF	TASK‐4 GOF leads to Na channel LOF	Moderate	NA
*TRPM4*	TRPM4	AD	BrS, Familial SSS	TRPM4 GOF reduce Na current	Low	NA/major gene for SSS
*HCN4*	HCN4	AD	LVNC, Familial SSS	?	Low	NA/major gene for SSS
2. Gap junction						
*GJA5*	Cx40	AD	Familial AF, atrial standstill	HPS conduction disturbance	High	NA/rare gene
*GJC1*	Cx45	AD	Familial AF, atrial standstill	AV node, atrial conduction disturbance	Low	NA/rare gene
3. Cardiomyopathy						
*LMNA*	Lamin A/C	AD, AR	Emery‐Dreifuss muscular dystrophy Type 2 (AD), 3 (AR), limb‐girdle muscular dystrophy, Hutchinson‐Gilford progeria syndrome, Werner's syndrome, Charcot–Marie–Tooth disease Type 2B1	Nuclear structure	Very high^b^ (SCD)	NA/major gene Definite for DCM
*EMD*	Emerin	XR	Emery‐Dreifuss muscular dystrophy Type 1	Nuclear Membrane	High	NA/rare gene
*DES*	Desmin	AD	Desminopathy	Accumulation of desmin	High	NA/rare gene Definite for DCM, moderate for ACM
*DMD*	Dystrophin	XR	DCM (CMD3B), muscular dystrophy (Becker or Duchenne type)	Fibrosis of HPS	Moderate	NA/rare gene
*TNNI3K*	TNNI3K	AD	DCM	?	Low	NA/rare gene
*DMPK*	DMPK	AD	Myotonic dystrophy (DM1)	Down‐regulation of Cx40 and Cx43	Moderate	NA/rare gene
*ZNF9*	CZNP	AD	DCM, myotonic dystrophy (DM2)	?	Low	NA/rare gene
4. Transcription factor						
*NKX2‐5*	NKX2‐5		ASD	Small AV node	Low	NA/rare gene
*TBX5*	Tbx5	AD	Holt‐Oram syndrome	Loss of fast conduction of AV node	Low	NA/rare gene
5. Metabolic disease						
*PRKAG2*	PRKAG2	AD	HCM, WPW	Metabolic^a^	High	NA/rare gene; definite for HCM
*LAMP2*	LAMP2B	XD	Danon disease	Metabolic^a^	Moderate	NA/rare gene Definite for HCM
*GLA*	α galactosidase	XD	Fabry disease	Metabolic^a^	Moderate	NA/rare gene; definite for HCM
6. Mitochondria disease						
mtDNA deletion		MI	Kearns‐Sayre syndrome, CPEO, MELAS, Leigh syndrome, MERRF	Loss of energy provide	High^b^	NA/rare gene

*Note:* 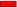 Grade 1: Core genes; 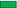 Grade 2: Established genes but less frequent; 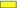 Grade 3: Emerging/research‐level genes.

Abbreviations: AD, autosomal dominant; AF, atrial fibrillation; AR, autosomal recessive; ARM, arrhythmogenic cardiomyopathy; ASD, atrial septal defect; BrS, Brugada syndrome; ClinGen, Clinical Genome Resource of NCBI, https://clinicalgenome.org; CMD3B, cardiomyopathy 3B; CPEO, chronic progressive external ophthalmoplegia; Cx40, connexin 40; Cx45, connexin 45; CZNP, Zink finger protein 9; DCM, dilated cardiomyopathy; DES, desmin; DMPK, DM1 protein kinase; EMD, emerin; GJA5, gap junction protein alpha 5; GJC1, gap junction protein gamma 1; GLA, galactosidase alpha; GOF, gain of function; HCM, hypertrophic cardiomyopathy; HCN4, hyperpolarization activated cyclic nucleotide gated potassium channel 4; HF, heart failure; HPS, His Purkinje system; KCNK17, TASK‐4, potassium two pore domain channel subfamily K member 17; LAMP2, lysosome associated membrane protein 2; LMNA, lamin A/C; LOF, loss of function; LQT2, Long QT syndrome type 2; LQT3, long QT syndrome type 3; LVH, left ventricular hyperttophy; LVNC, left ventricler noncompaction; MELAS, mitochondrial myopathy, encephalopathy, lactic acidosis, stroke‐like episodes; MERRF, myoclonic epilepsy with ragged red fibers; MI, mitochondrial inheritance; NA, not available, or not yet curated; NKX2‐5, NK2 homeobox 5; PRKAG2, protein kinase AMP‐activated non‐catalytic subunit gamma 2; SCD, sudden cardiac death; SCN5A, NaV1.5, sodium voltage‐gated channel alpha subunit 5; SSS, sick sinus syndrome; TBX5, T‐box transcription factor 5; TNNI3K, TNNI3 interacting kinase; TRPM4, transient receptor potential cation channel subfamily M member 4; VF v = ventricular fibrillation; WPW, Wolff–Parkinson‐White syndrome; XD, X‐linked dominant; XR, X‐linked recessive.

^a^ Infiltration of substance into the conduction system.

^b^ Risks for unexpected death was high but independent of PCCD.

### 8.1 Grade 1 Core Genes

These genes have high clinical relevance and should be systematically tested to find the cause of PCCD.

### 8.2 Grade 2 Established Genes but Less Frequent

These genes have been established to cause PCCD, but are less frequent than core genes, and often are associated with mixed phenotypes or structural/developmental abnormalities, such as cardiomyopathies or congenital heart disease.

### 8.3 Grade 3 Emerging/Research‐Level Genes

Some of these gene variants are known to cause PCCD, but the diagnostic value is less than that for Grade 1 and Grade 2 genes. These genes are not included in routine clinical testing for diagnosis of PCCD.

## 9 Diagnostic Approach

We propose a structured clinical workflow for the diagnosis of PCCD.

### 9.1 Exclude Known Causes of Conduction Disease

The first step is to rule out known causes that may lead to conduction disturbances. Patients diagnosed with long QT syndrome associated with functional AV block or short QT syndrome should not be included in PCCD. Patients with BrS may overlap with PCCD when the *SCN5A* variant phenotype causes a conduction disease. In the case of congenital AV block in mothers with anti‐SS‐A/SS‐B antibodies, ischemia of the AV nodal branch artery, myxoma or metastatic tumor around the AV nodal region, sarcoidosis, myocarditis caused by a virus (coxsackie, adeno, influenza, COVID‐19, etc.) or other pathogens (Lyme disease, Chagas disease, diphtheria, etc.), rheumatic fever, infectious endocarditis, aortic valve replacement (surgical or transcatheter aortic valve implantation), mitral valve replacement, surgical closure of atrioventricular septal defect, congenital corrected transposition of the great arteries, polysplenia, and catheter ablation near the AV node have the potential risk of causing conduction disturbances and should be ruled out.

### 9.2 Exclude Reversible Causes

The second step is to confirm the conduction disturbance is not transient or reversible. Wenckebach AV block, vasovagal reflex, slow AV nodal conduction, hyperkalemia, or medications that prolong AV conduction, such as ß blockers, Ca antagonists (verapamil, diltiazem), digoxin, and antiarrhythmic agents (amiodarone, etc.) should be ruled out.

### 9.3 Evaluation

Age at the onset is essentially important. Cardiac conduction disturbances are a part of the aging process; therefore, the onset of a conduction disturbance at a younger age (< 50 years) is valid for a diagnosis of PCCD.

If young patients have a family history of PMI, conduction disturbance, bradycardia, or SCD, it may help suggest disease inheritance, but some PCCD may have no family history and may be a de novo occurrence.

One should check for associated phenotypes, mental retardation, epilepsy, autism, muscle weakness, existence of external ophthalmoplegia, radial deformities, syndactyly, HCM, DCM, and ASD. These phenotypes may lead to the diagnosis of some syndrome.

### 9.4 Perform Genetic Testing

Finally, perform genetic testing targeting the most probable gene based on the documented phenotype, or begin with the Grade 1 genes we have suggested. Because many syndromes or diseases cause PCCD, genetic testing was not performed for a definite diagnosis of PCCD, but the results led to a diagnosis of some syndrome or disease that may cause PCCD.

### 9.5 Proposal of Diagnostic Criteria

From the experience obtained in our cases, we propose diagnostic criteria for PCCD (Table 2). The total score was added up in the absence of exclusion criteria. According to these criteria, PCCD was defined as having high probability (≥ 3.5 points), intermediate probability (1.5–3 points), or low probability (≤ 1 point). This proposed scoring criteria were strongly based on the experience of the authors; however, this score is exploratory and has not yet been validated.

**TABLE 2:** Proposal of diagnostic criteria for PCCD.

	Points
Electrocardiogram^a^	
A. AV block	
Mobitz type II, paroxysmal, or complete AV block	2
I° AV block (PR > 200 ms)	0.5
B. QRS duration	
≥ 20 ms increase from previous QRS duration^b^ or	2
QRS > 120 ms	1
C. HV block or HV > 55 ms	0.5
Clinical	
A. Syncope or Dizziness	
With bradycardia or cardiac arrest	1
Ventricular fibrillation	0.5
B. Onset age < 50 years	0.5
Familial history	
A. AV block or pacemaker implantation < 50 years	1
B. Sudden cardiac death < 30 years	0.5

^a^ Exclusion criteria: long QT syndrome, short QT syndrome, Brugada syndrome (some SCN5A variants may overlap with PCCD), congenital AV block from mothers with anti‐SS‐A/SS‐B antibody, ischemia of AV nodal branch artery, myxoma or metastatic tumor around AV nodal region, sarcoidosis, myocarditis by virus (coxsackie, adeno, influenza, COVID‐19, etc.) or other pathogen (Lyme disease, Chagas disease, diphtheria, etc.), rheumatic fever, infectious endocarditis, aortic valve replacement (surgical or transcatheter aortic valve implantation), mitral valve replacement, surgical closure of atrioventricular septal defect, congenital corrected transposition of the great arteries, polysplenia, catheter ablation near AV node through slow pathway, Wenckebach AV block, vasovagal reflex, slow AV nodal conduction, hyperkalemia, or medications to prolong AV conduction, such as ß blocker, Ca antagonist (verapamil, diltiazem), digoxin, and antiarrhythmic agent (amiodarone, etc.).

^b^ Intermittent or rate dependent bundle branch block should be excluded ≥ 3.5: high probability; 1.5–3: intermediate probability; ≤ 1: low probability.

## 10 Management of PCCD

### 10.1 Device Implantation

Not all PCCD patients have the same risk profile, so risk stratification is essential, and management should integrate genetic information. According to the expert consensus statement [54], device implantation for PCCD is recommended as follows.

#### 10.1.1 Class I

1. Pacemaker implantation is recommended in patients with a diagnosis of PCCD and the presence of
  - Intermittent or permanent third‐degree or high‐grade AV block or
  - Symptomatic second‐degree AV block (Mobitz I or II).

#### 10.1.2 Class IIa

2 Pacemaker implantation can be useful in patients with a diagnosis of PCCD and the presence of bifascicular block with or without first‐degree AV block.
3 Implantable cardioverter defibrillator (ICD) implantation is advised in adult patients diagnosed with PCCD with a pathogenic variant in the lamin A/C gene with left ventricular dysfunction and/or nonsustained VT.

Although there was no recommendation for the management of PCCD, ESC guidelines noted that permanent pacemaker implantation may also be considered in patients with Kearns‐Sayre syndrome, Emery‐Dreifuss, or limb‐girdle muscular dystrophy with any degree of AV block because of the substantial risk of rapid progression to total block [55]. In patients with limb–girdle type 1B or Emery‐Dreifuss muscular dystrophies who have an indication for pacing or significant late gadolinium enhancement on cardiac magnetic resonance, ICD implantation should be considered. The following indications of ICD were recommended related to the PCCD.

#### 10.1.3 Class I

1. ICD implantation is recommended in patients with myotonic dystrophy and sustained monomorphic VT or aborted cardiac arrest not caused by bundle branch re‐entrant VT.
2. ICD implantation is recommended in patients with BrS who:
  - Are survivors of an aborted cardiac arrest and/or
  - Have documented spontaneous sustained VT

#### 10.1.4 Class IIa

1. In myotonic dystrophy patients without AV conduction delay and a syncope highly suspicious for ventricular arrhythmias, ICD implantation should be considered.
2. In myotonic dystrophy patients with palpitations highly suspicious for ventricular arrhythmias and induction of a non‐bundle branch re‐entrant VT, ICD implantation should be considered.
3. In patients with limb‐girdle type 1B or Emery‐Dreifuss muscular dystrophies and indication for pacing, ICD implantation should be considered.
4. ICD implantation should be considered in patients with type 1 Brugada pattern and an arrhythmic syncope.

### 10.2 Pacemaker Implantation

PMI is indicated in symptomatic AV block or high‐grade conduction disease. Although PMI improves the survival rate and reduces morbidity, a certain number of patients with PCCD have the risk of sudden cardiac death (SCD), possibly due to VT or ventricular fibrillation (VF). PMI is also useful for continuous monitoring of the disease progression and ventricular arrhythmia occurrence.

### 10.3 ICD Implantation

To date, loss‐of‐function variants in *SCN5A*, as well as variants in *DES and LMNA* [56], and mitochondrial diseases [12], have been associated with a high risk of SCD, and ICD implantation should be considered. We propose ICD implantation in patients with the following variants when the patients have PCCD and have episodes of ventricular arrhythmias:

- *LMNA* variant carriers with left ventricular dysfunction
- *DES*‐related cardiomyopathy
- *SCN5A* variant when the patients were survivors of cardiac arrest or had spontaneous sustained VT
- Mitochondrial disease

### 10.4 Pharmacological Treatment

Active treatment with antipyretics for fever in patients with *SCN5A‐*mediated PCCD is recommended to prevent fever‐induced ventricular arrhythmias [54], as well‐established in BrS. Cilostazol and milrinone are phosphodiesterase (PDE) inhibitors and increase contractility of the cardiac muscle and heart rate through an increase in cAMP. These actions have been reported to be beneficial for BrS [57], but there has been no evidence for their effectiveness for conduction defects.

## 11 Conclusion

PCCD is a rare inheritable cardiac disease that may lead to complete heart block, syncope, or sudden cardiac death. Many varieties of genes, such as ion channel genes, gap junction genes, genes associated with myopathy, transcription factor genes, genes associated with metabolic disease, and mitochondrial gene variants have all been associated with the occurrence of PCCD. However, definitive diagnostic criteria for PCCD have not yet been established, likely because there is no consensus regarding which disease entities should be included in the diagnosis of PCCD. We recommend including a wide variety of diseases within the diagnostic spectrum of PCCD. Pacemaker implantation is the first‐line therapy for PCCD, but ICD implantation should be considered in some cases.

## Disclosure

Limitations: The proposed diagnostic criteria are exploratory and have not yet been validated; therefore, they require further refinement in future studies.

## Ethics Statement

This clinical review was conducted based on previously published case reports and guidelines.

## Consent

The authors have nothing to report.

## Conflicts of Interest

The authors declare no conflicts of interest.

## Data Availability

The data that support the findings of this study are available from the corresponding author upon reasonable request.
